# Persistent fire foci in all biomes undermine the Paris Agreement in Brazil

**DOI:** 10.1038/s41598-020-72571-w

**Published:** 2020-10-01

**Authors:** Carlos Antonio da Silva Junior, Paulo Eduardo Teodoro, Rafael Coll Delgado, Larissa Pereira Ribeiro Teodoro, Mendelson Lima, Ariane de Andréa Pantaleão, Fabio Henrique Rojo Baio, Gileno Brito de Azevedo, Glauce Taís de Oliveira Sousa Azevedo, Guilherme Fernando Capristo-Silva, Damien Arvor, Cassiele Uliana Facco

**Affiliations:** 1 Department of Geography, State University of Mato Grosso (UNEMAT), Sinop, Mato Grosso 78555000 Brazil; 2 Federal University of Mato Grosso do Sul (UFMS), Chapadão do Sul, Mato Grosso do Sul 79560-000 Brazil; 3 Department of Environmental Sciences, Forest Institute, Federal Rural University of Rio de Janeiro (UFRRJ), Seropédica, Rio de Janeiro 23897-000 Brazil; 4 State University of Mato Grosso (UNEMAT), Alta Floresta, Mato Grosso 78580000 Brazil; 5 Postgraduate Program in Agronomy, Federal University of Mato Grosso (UFMT), Sinop, Mato Grosso 78555000 Brazil; 6 CNRS, UMR 6554 LETG, Université Rennes 2, 35043 Rennes, France; 7 Postgraduate Program in Management and Regulation of Water Resources - ProfÁgua, State University of Mato Grosso (UNEMAT), Cuiabá, Mato Grosso Brazil

**Keywords:** Environmental sciences, Climate sciences

## Abstract

Brazil is one of the world’s biggest emitters of greenhouse gases (GHGs). Fire foci across the country contributes to these emissions and compromises emission reduction targets pledged by Brazil under the Paris Agreement. In this paper, we quantify fire foci, burned areas, and carbon emissions in all Brazilian biomes (i.e., Amazon, Cerrado, Caatinga, Atlantic Forest, Pantanal and Pampa). We analyzed these variables using cluster analysis and non-parametric statistics to predict carbon and CO_2_ emissions for the next decade. Our results showed no increase in the number of fire foci and carbon emissions for the evaluated time series, whereby the highest emissions occur and will persist in the Amazon and Cerrado biomes. The Atlantic Forest, Pantanal, Caatinga and Pampa biomes had low emissions compared to the Amazon and Cerrado. Based on 2030 projections, the sum of emissions from fire foci in the six Brazilian biomes will exceed 5.7 Gt CO2, compromising the national GHG reduction targets. To reduce GHG emissions, Brazil will need to control deforestation induced by the expansion of the agricultural frontier in the Amazon and Cerrado biomes. This can only be achieved through significant political effort involving the government, entrepreneurs and society as a collective.

## Introduction

The 2015 Paris Agreement was the result of a worldwide effort to combat global warming. It came into effect in 2016 and Brazil committed to a target of reducing its greenhouse gas (GHG) emissions to 43% of its 2005 level by 2030^1^. At the time, Brazil presented promising results in terms of reducing deforestation in the legal Amazon area by 67.35% (from 19,014 km^2^ in 2005 to 6207 km^2^ in 2015). However, since this time, the situation has rapidly evolved; from 2018 to 2019, deforestation in this region has increased by 30% (from 7536 to 9762 km^2^)^2^. As such, Brazil still remains the seventh biggest emitter of GHGs in the world with 1939 billion gross tons of GHGs emitted in 2018^3^. Of these emissions, 69% were due to land use changes, particularly relating to agriculture and deforestation^3^; the Amazon and Cerrado biomes experienced the highest rates of deforestation. With deforestation comes the occurrence of fires, considered the cheapest and most effective way to clean up deforested areas or to clean up degraded areas, and are widely practiced throughout the country. Fires in Brazil are mostly of anthropogenic origin and affect the distribution of ecosystems, compromising the reproduction and survivability of plant and animal species^4^, in addition to its impacts on the carbon cycle and global climate^5^.

In 2019 alone, Brazil had 197,632 fires distributed across the country^6^. These fires were spread across all the distinct biomes (i.e., Amazon, Cerrado, Atlantic Forest, Pantanal, Caatinga and Pampa), with differing degrees of intensity and a substantial GHG contribution to the atmosphere. Some of these differences have been attributed to years of climate severity in some regions of the country. These have been directly associated with the El Niño-Southern Oscillation (ENSO) and Sea Surface Temperature (SST) anomalies of the Atlantic highlight to super denomination El Niño^7–9^.

Brazil has already shown that it is possible to reduce deforestation and its GHG emissions^10,11^. However, the synergies between new environmental policies^12^ and the return of deforestation, numerous fires and the extreme climate-related events that may occur, threaten to compromise the country’s GHG emission reduction commitments under the Paris Agreement. In this paper, we quantify fire foci and their emissions in all Brazilian biomes between 1999 and 2018, and present future emissions projections for 2030. This study aims to address five key questions:

(i) do fire foci have a direct relationship with CO_2_ emissions observed in recent years?
(ii) what is the contribution of each biome to the total Brazilian GHG emissions?
(iii) what is the projected GHG emissions in 2030 with the implementation of current environmental policies?
(iv) what are the key biomes in Brazil that need to reduce their emissions in order to achieve the emission reduction targets as per the 2015 Paris Agreement?

An understanding of the spatio-temporal distribution of fire foci in Brazil will enable safe planning of agricultural zoning, urban growth, commerce, industrial activities, tourism, and civil defense. It will also inform the development of public policies targeted toward the conservation of biomes and strategies to reduce GHG emissions in the country.

## Results

### Descriptive statistics applied to fire foci and carbon emissions

Between 1999 and 2018, 16,141,383 fire foci were detected across Brazil; the most affected biomes were the Cerrado and Amazon which constituted 41.56% and 38.34% of total fire foci, respectively. In addition, both biomes consisted of similar seasonal patterns with fire peaks from June to September (see Supplementary Fig. S1). Approximately 9.89% of fires occurred in the Atlantic Forest, largely between January and April. Of the total fire foci, 5.94% were located in the Caatinga biome, especially in November and December. The biomes with the least number of fire foci were the Pantanal and Pampa biomes accounting for 3.83% and 0.44% of the total, respectively. Regardless of the month and biome, outliers were also present in the time series.

Fires in all Brazilian biomes produced of 8089.17 Tg of carbon emissions between 1999 and 2018. Most emissions originated from the Amazon (60.71%) and Cerrado biomes (32.04%), whilst the contribution from other biomes was less than 3% each. The highest monthly carbon emissions were measured in the Amazon (see Supplementary Fig. S2), although the Cerrado also had significant emissions between April and October.

**Figure 1:**
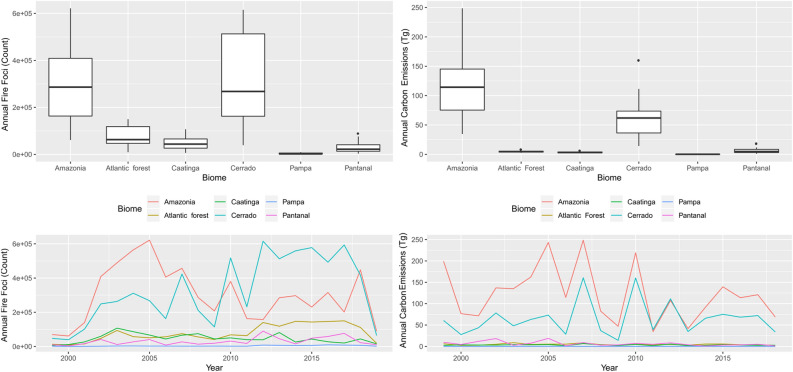
Boxplot applied to annual fire foci and carbon emission (Tg) and temporal evolution of these variables between 1999 and 2018. Ggplot package of R (v3.2.1, https://cran.r-project.org/web/packages/ggplot2/index.html) was used to create the line and boxplot graphics.

Until 2009, the annual evolution of fire foci occurred most frequently in the Amazon (Fig. 1). However, from 2010 onwards, the situation evolved as a significant number of fires were located in the Cerrado. Regardless, the Amazon continued to be the main source of carbon emissions.

### Temporal patterns for annual fire foci and carbon emissions

**Figure 2:**
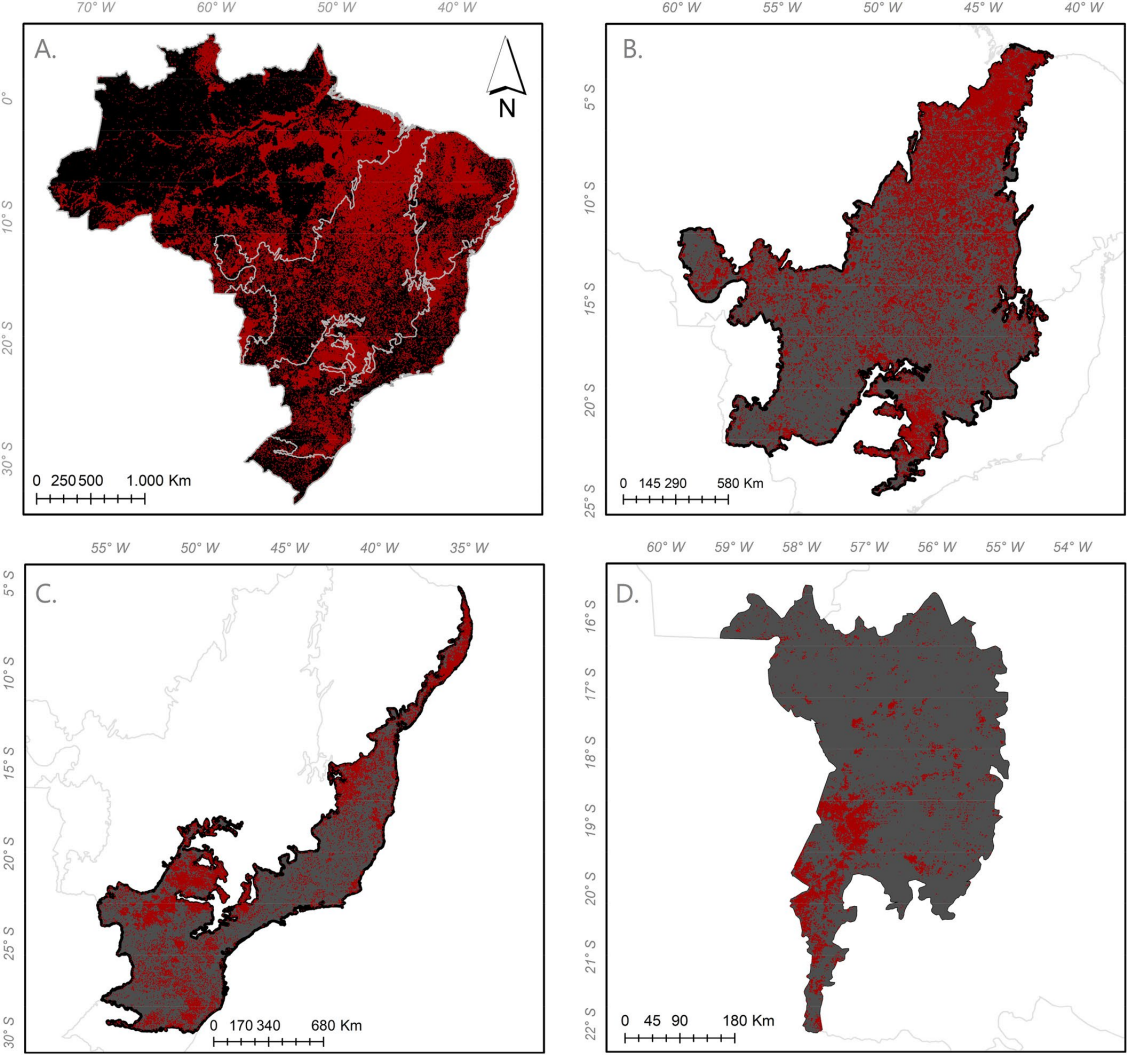
Fire foci in Brazilian biomes in 2009, where (**A**) delimitation of biomes in Brazil; (**B**) Cerrado; (**C**) Mata Atlântica; (**D**) Pantanal.

**Figure 3:**
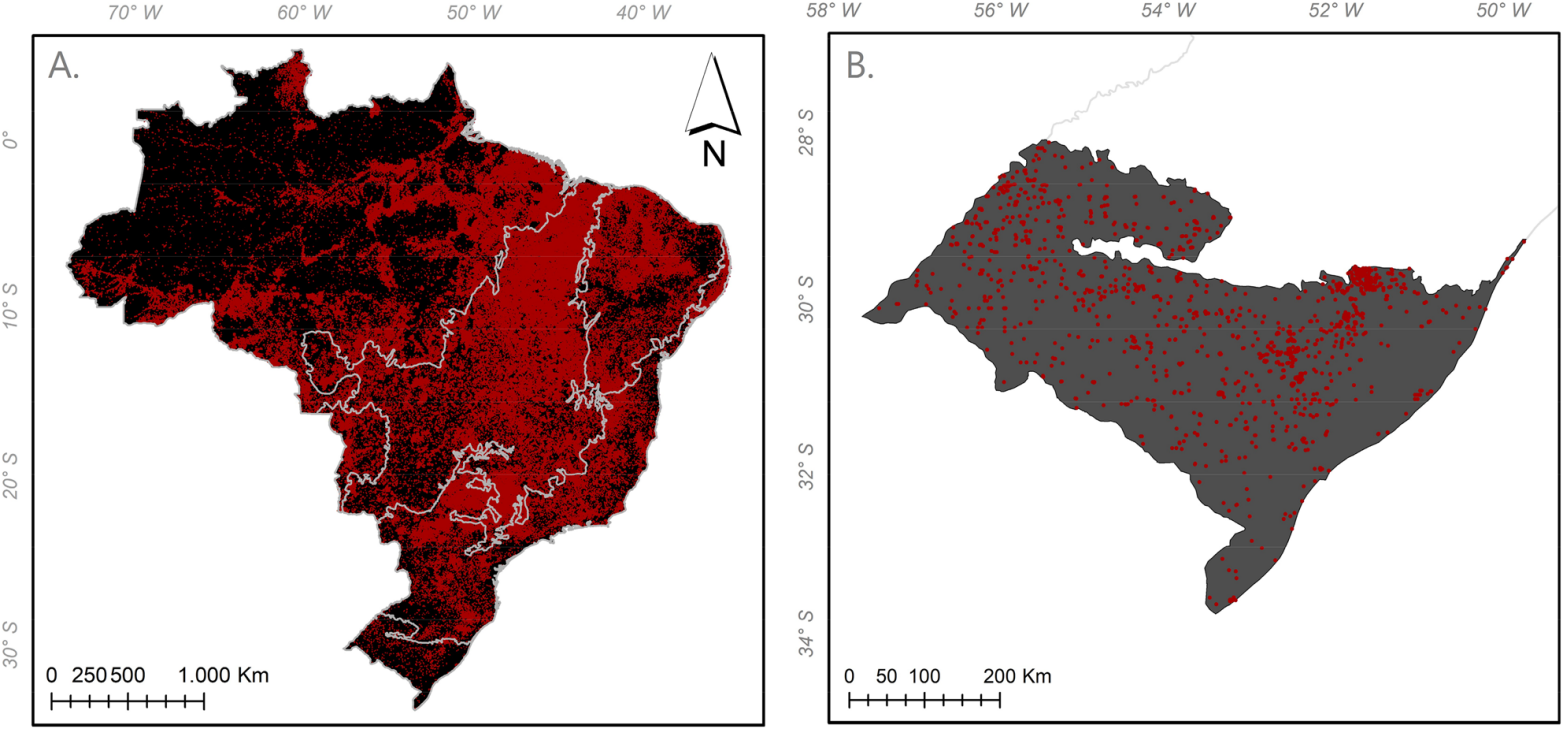
Fire foci in Brazilian biomes in 2011, where (**A**) delimitation of biomes in Brazil; (**B**) Pampa.

**Figure 4:**
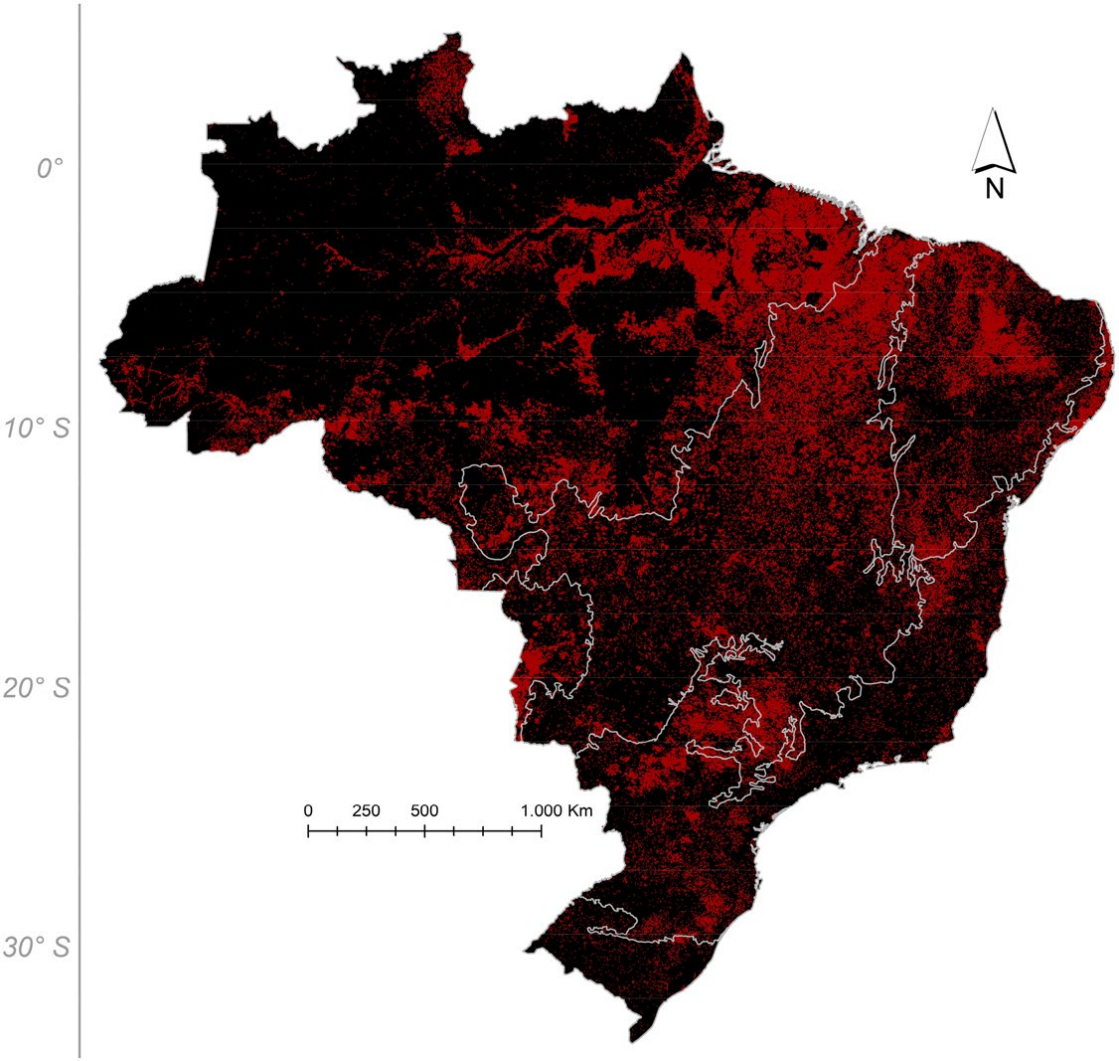
Fire foci in Brazilian biomes in 2018.

Trend analysis was carried out for data on the annual fire foci and carbon emissions (Table 1). The Man-Kendall test highlighted a significant upward trend in fire foci for the Atlantic Forest, Cerrado, Pantanal and Pampa biomes. The Pettitt test identified turning points in the fire foci patterns for the Atlantic Forest, Cerrado and Pampa biomes in 2009, 2009 and 2011, respectively. To illustrate this result, we mapped the spatial distribution of fires for 2009 (Fig. 2), 2011 (Fig. 3), and 2018 using the Moderate Resolution Imaging Spectroradiometer (MODIS) sensor (Fig. 4). Note that the 2018 dataset was used to demonstrate a more recent situation).

**Table 1:** P-value of Mann–Kendall trend test and Pettitt test applied to annual fire foci and Carbon emissions.

Biome	Fire foci			Carbon emissions		
	Mann–Kendall	Pettitt	Year	Mann–Kendall	Pettitt	Year
Amazonia	0.65	0.37	–	0.31	0.27	–
Atlantic Forest	0.01	0.02	2009	0.35	0.67	–
Caatinga	0.26	0.42	–	0.72	0.96	–
Cerrado	0.01	0.04	2009	0.67	0.19	–
Pampa	0.01	0.03	2011	0.92	0.64	–
Pantanal	0.04	0.05	2009	0.06	0.20	–

**Figure 5:**
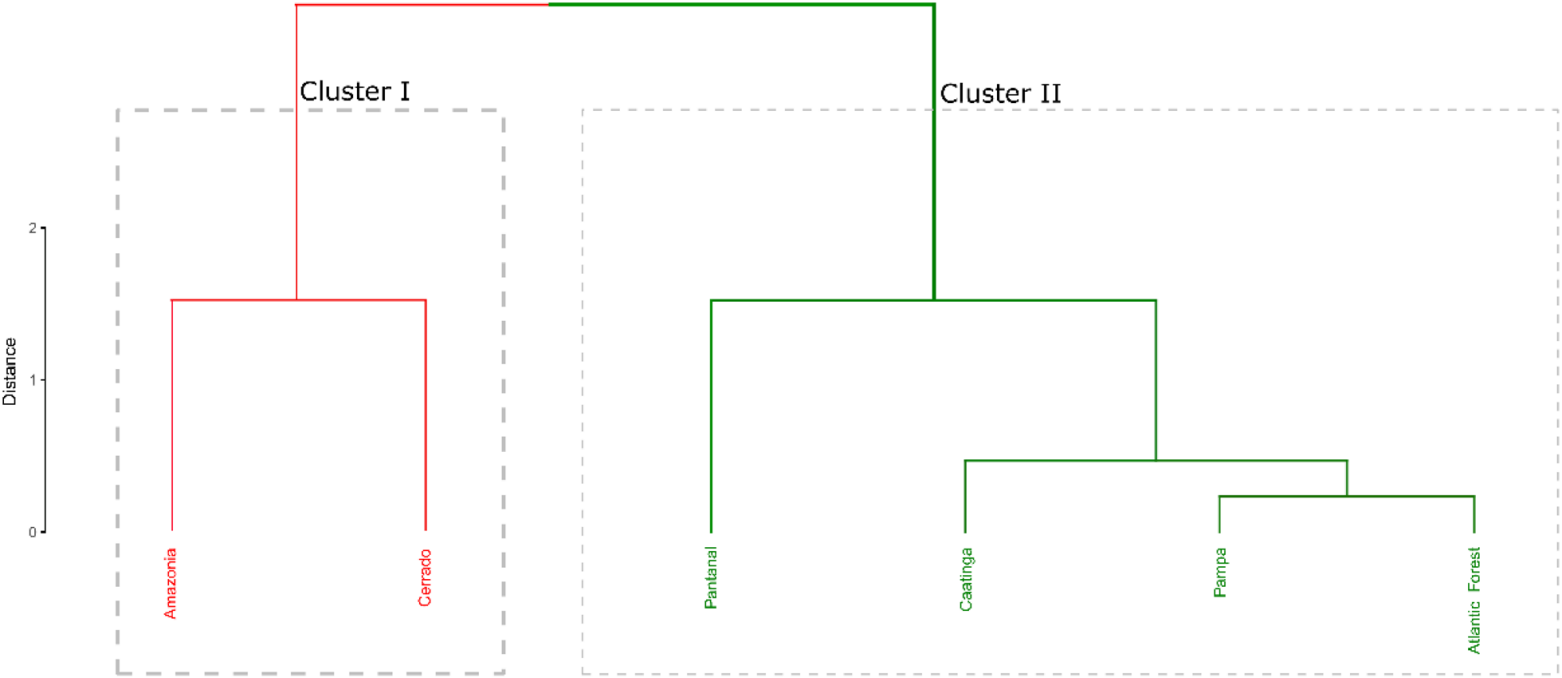
Cluster analysis applied to Brazilian biomes using annual carbon emission and fire foci. Package used of R to create the cluster were ggplot (v3.2.1, https://cran.r-project.org/web/packages/ggplot2/index.html) and factoextra (v1.0.7, https://cran.r-project.org/web/packages/factoextra/index.html).

The cluster analysis in Fig. 5, shows the formation of two homogeneous groups; the first containing the Amazon and Cerrado biomes, and the second containing the Atlantic Forest, Caatinga, Pantanal and Pampa biomes.

### Model validation and projected fire foci and carbon emissions in Brazilian biomes

**Figure 6:**
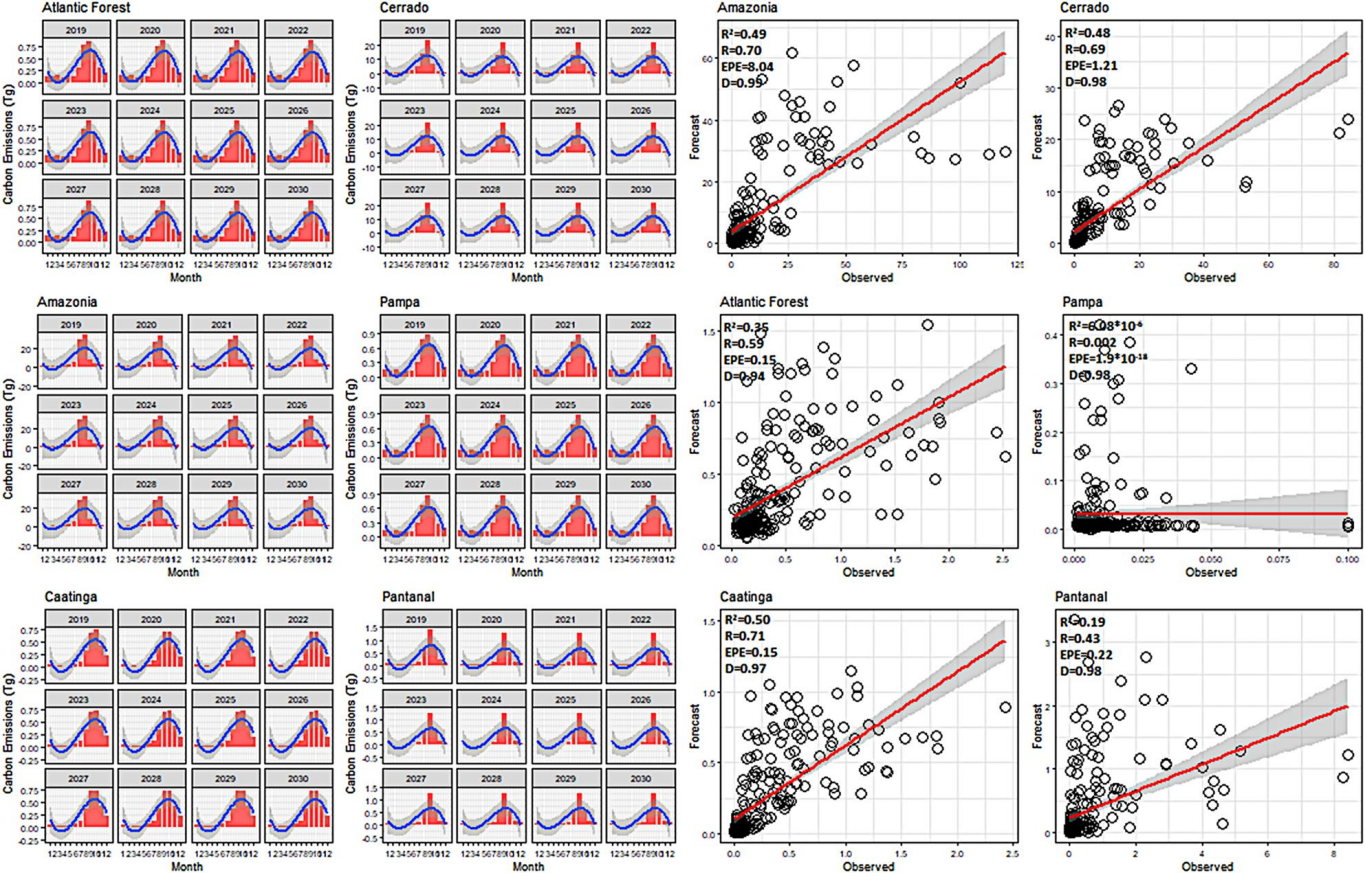
ARIMA modeling and validation for carbon emissions in the Brazilian biomes. Package used of R to create the line, bar and regression graphs were ggplot with multiplot (v3.2.1, https://cran.r-project.org/web/packages/ggplot2/index.html).

**Figure 7:**
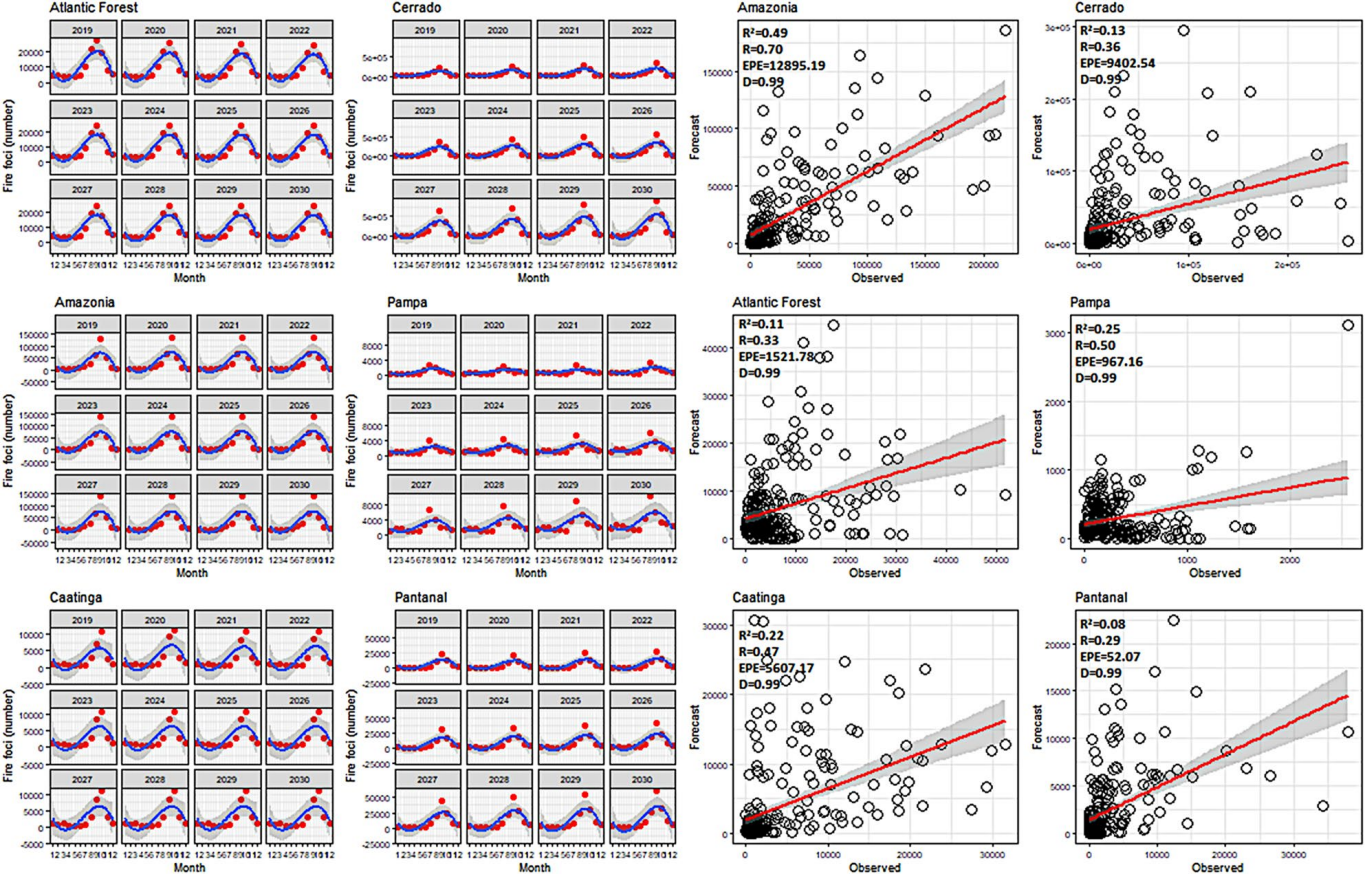
ARIMA modeling and validation for fire foci in the Brazilian biomes. Package used of R to create the line, bar and regression graphs were ggplot with multiplot (v3.2.1, https://cran.r-project.org/web/packages/ggplot2/index.html).

Based on statistical analyses used to validate the models developed for carbon emissions and fire foci, the most optimum coefficients of determination (*R*^2^) for carbon emissions were for Caatinga (0.50), Amazon (0.49), and Cerrado (0.48) (Fig. 6). A poor correlation was observed in the Pampa and Pantanal biomes; the Willmott (D) precision index was above 0.9 (Fig. 6). Despite these poor correlations for some biomes, regression analysis showed the positive linear correlation of an increase in fire foci and carbon emissions across all biomes. The lowest errors were associated with Pampa (1*.*9 × 10^*−*18^ Tg CO_2_ emissions), followed by the Atlantic Forest (0.15 Tg CO_2_ emissions) and Caatinga (0.15 Tg CO_2_ emissions). The largest and most significant error was associated with the Amazon with carbon emissions of 8.04 Tg, respectively. The predictions indicate that carbon emissions will increase in the Amazon and Cerrado. From 2019 to 2030, the highest emissions for the Amazon and Cerrado are projected to occur in September, with values exceeding 33 and 21 Tg, respectively (Fig. 7). The lowest emissions are predicted to occur in Caatinga, Pampa and Pantanal.

**Figure 8:**
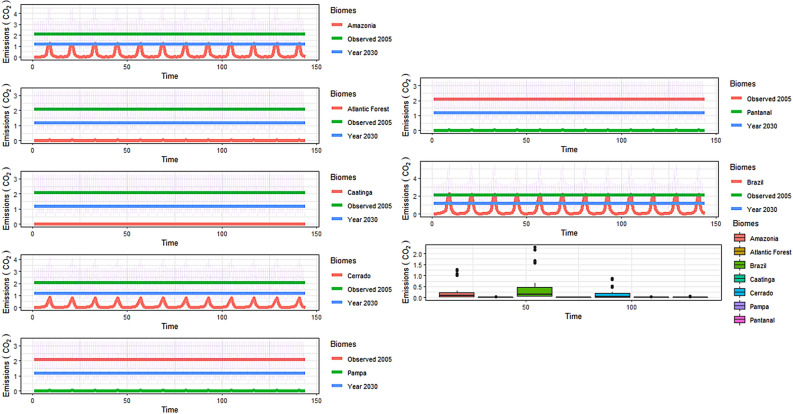
Predictions of *CO*_2_ emissions for the 2019–2030 time period and for all Brazilian biomes. Package used of R to create the line and boxplot graphs were ggplot with multiplot (v3.2.1, https://cran.r-project.org/web/packages/ggplot2/index.html).

In the analysis of past validations of fire foci, the highest correlation was observed for the Amazon (0.70) and the lowest for Pantanal (0.29). In precision coefficient analysis, the "D" index was high alongside the carbon emissions, where index values were above 0.9 (Fig. 8). The largest fire foci error was present in the Amazon experiencing greater than 12,000 fire foci, whilst the smallest error was present in Pantanal with a record of 52.07 fire foci (Fig. 7). In terms of predicted future fire foci for all biomes, the Cerrado was projected to experience a significant increase, with greater than 900,000 fire foci predicted for September 2030. This was followed by the Amazon which was predicted to experience 143,000 fire foci in September 2030 (Fig. 7). The Pampa and Pantanal were projected to experience the lowest fire foci.

In the conversion of carbon emissions into CO_2_ emissions (Fig. 8), the most significant emissions originated from the Amazon and Cerrado, with emissions exceeding 1.23 and 0.87 Gt CO_2_ in September. The Amazon and the Cerrado also had the largest CO_2_ emission means and outliers. The lowest CO_2_ emissions occurred in the Atlantic Forest, Pantanal, Pampa and Caatinga. In terms of the analysis of CO_2_ emissions in 2005 compared to the target for 2030, all biomes, with the exception of the Amazon, generated lower emissions in the latter year than those measured in 2005. However, for the Amazon, the CO_2_ emissions were greater than the 2030 target (Fig. 8). In the integrated data analysis for all biomes, the most critical month was September, where there is an increase in fire foci and carbon emissions, and a considerable increase in CO_2_ emissions that exceed 2.1 Gt CO_2_ (Fig. 8).

## Discussion

### Fire foci and carbon emissions

Only 7.6% of Brazil’s total land mass (approximately 8,511,000 km^2^) is utilized for agriculture to cultivate major crops such as soybean, corn, cotton, sugarcane and irrigated rice^13^, as well as agriculture for extensive cattle ranching^14^. Brazil benefits from diverse soil and climatic conditions that are distributed among the six biomes. These biomes are prone to accidental fires and those that may occur as a result of inappropriate management practices^14,15^. Fires and the burning of biomass impacts ecosystem processes across a wide range of biomes at the regional and global scales^16^.

In this study, fires and carbon emissions (atmospheric CO_2_) measured between 1999 and 2018 predominately originate from the Amazon. In a recent study the links between fire foci and meteorological variables were related to the ENSO in the Amazonian state of Acre. Da Silva Junior et al.^9^ emphasized a significant increase in the number of fires during the Neutrality phase as well as an increase in burned areas during the La Niña phase. This sudden change in fire foci frequency during Neutrality and the links between the most significantly burned areas during the La Niña phenomenon and not the El Niño event, may be associated with accelerated logging. This is particularly the case for the regions with Brazil nut forests, that are currently today considered the most profitable extractive sector for Acre^9^.

In addition to the high relevance of accelerated landscape changes from anthropogenic causes for fire foci, some authors have correlated the increase in fires in Amazon as a result of the changes to atmospheric circulation and increases in the North Atlantic Ocean Surface temperatures^17^.

It is also important to consider the size of the affected area where an increase in fires is associated with coverage and a portion of the Amazon in Brazilian territory (greater than 49%). In a study on fires in Brazil, Caúla et al.^18^ analyzed the relationship between the number of fires and the burned area. They concluded that the burned area does not always reflect the concentration of fires when area density and fire foci are used. Researchers have also observed an increase in the number of fires during winter and spring, consistent with the findings from this study.

Another recent study in the state of Amazonas applied land use and occupation procedures, meteorological variables and the density of fire foci^7^. They found that forest cover experienced the most significant number of fire occurrences as this was a region with the largest largest forested area of forest when compared to regions^7^.

It is also necessary to associate extreme weather events such as the significant droughts that occurred in 2005, 2010 and 2015, mainly in Amazonas, alongside the incremental number of fires related to the burned areas. Barbosa et al.^7^ used the Kernel Density spatial technique during the rainy season in 2005 for Amazonas to verify the dramatic increase in fires concentrated in the forested regions of Igapó, Várzea and Terra Firme, and in pastures and near roads. There was also a significant increase in the number of fires during winter and spring.

The acceleration of deforestation in the Amazon is associated with human activities and agricultural expansion in this region. Deforestation due to human activities highlights the concern for future generations regarding atmospheric carbon emissions^19^. As the Amazon possesses the largest national-territorial portion, there were higher carbon emissions throughout the time series (Fig. 2); this is a major concern for high atmospheric carbon concentrations. In this study, 2007 had the highest carbon emissions, whilst Aragão et al.^19^ found that the highest emissions occurred during the 2015 dry season. This difference may be associated with the territorial portion that was used.

It is also necessary to determine the temporal carbon emission and fire trends in order to adopt strategies to reduce forest fires, and carbon emissions. We observed a significant trend mainly for the Pampa, Cerrado, and the Atlantic Forest biomes. If more effective public policies are not implemented, Brazil is predicted to experience an increase in fires in these regions characterized by high biodiversity and stored carbon^20^. Studies in the Amazon, such as Fonseca et al.^15^, where future scenarios Representative Concentration Pathway (RCPs) were used, found a significant increase in the probability of fire in the Amazon, occurring mainly in October. These researchers reported that even under the most pessimistic scenarios, the potential to decrease fires in the Amazon would only occur with a drastic reduction in deforestation and the implementation of policies that aim to control and inspect protected areas in the Amazon.

Results from the Cerrado biome did not greatly deviate from the findings for the Amazon, as it was experienced the second highest number of fires in the time series. Several studies observed the resilience of this biome to withstand fires, especially in the driest phase. Trees are able to withstand large fires mainly due to their bark, which plays an important role in multiple physiological functions such as withstanding a significant fire event and storing water in its structure^21^.

Natural fires in Cerrado easily occurred due to lightning^22^. These fires usually occur during the rainy season, producing low-intensity fires that trigger a positive feedback loop by favoring the growth of grass species that is not to the detriment detriment of tree species^23^. However, fires resulting from anthropogenic activities are more intense and persist for a greater duration and occur mainly in the dry season. These fires trigger changes to the floristic composition and community structure of tree-shrub vegetation^24,25^. The occurrence of forest fires affects the accumulation of aerial woody biomass and causes a substantial reduction in the biomass of fire-sensitive species, transforms the Cerrado from a carbon sink to a source of CO_2_ emissions^25^.

The anthropogenic causes of fires in Cerrado may be attributed to cattle raising, where burning is a practice to restore pastures. In a study aiming to estimate GHG emissions from cattle raising in Brazil, Bustamante et al.^26^ reported that 36.8% of Brazil’s pastures are located in Cerrado (546,250.9 km^2^). They also verified that approximately 50% (1.69 Mt CO_2_ eq) of total CO_2_ emissions for pasture management in Brazil occurred in this biome.

**Figure 9:**
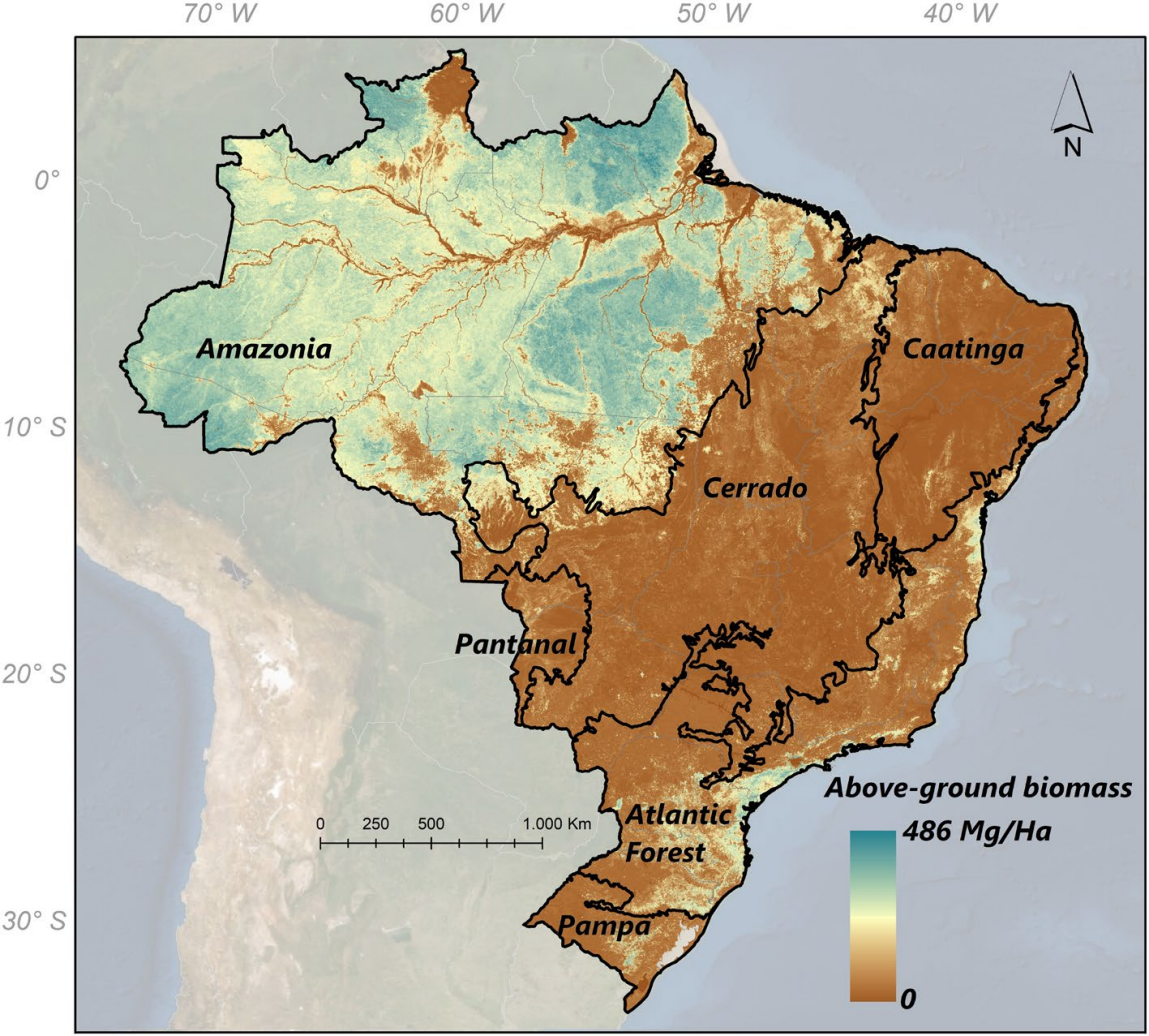
Distribution of Brazilian biomes across the national territory and estimates of above-ground biomass (in Mg/Ha). To prepare image was used the ArcGIS Pro with data Megagrams of aboveground live woody biomass per Hectare available through the Google Earth Engine platform (Google, https://earthengine.google.com/) through the dataset available at ee.Image ("www.WHRC/biomass/tropical").

This burning in the Cerrado has been accelerating and contributing to carbon emissions and the fire frequency in the Amazon (Fig. 4), due to the significant grouping with the Amazon (Fig. 9). As burning in this biome increases, it may significantly alter atmospheric chemistry, as reported in recent study on the Eastern Amazon and Cerrado; Pope et al.^27^ found an increase in burning rates, mainly between 2005 and 2016. This significant increase in fires as a result of deforestation in the Amazon and the increase in fires in the Cerrado produces atmospheric CO_2_ emissions to the troposphere and exacerbates the ozone problem, further accelerating global warming.

In the Atlantic Forest, the third-largest biome in Brazil, fires largely occurred during winter and spring; this was largely characteristic of the southeastern portion. Here, the semi-permanent high-pressure circulation of the South Atlantic is evident, featuring a drier landscape with a significant reduction in relative humidity and rainfall. In the Southeast region of the state of Rio de Janeiro, Andrade et al.^8^ also conducted a study on extreme drought events that occurred in 2005, 2010 and 2015 based on the use and occupation of soils, meteorological variables, and atmospheric circulation. They found a significant increase in the number of fires during winter and spring and associated the fire densities to the portion of the argisol and latosol soil classes in 2005 and 2010 and gleisol in 2015; these are most prevalent soil classes in Rio de Janeiro.

Although carbon emissions in the Atlantic Forest are relatively insignificant when compared to the Cerrado and Amazon, the trends observed as per the Mann–Kendall test highlights that there is a great concern for future generations of the Atlantic Forest, Cerrado, and Pampa biomes. In a study on the seasonality of Gross Primary Productivity (GPP) in the Itatiaia-Brazil National Park, Delgado et al.^28^ found an increase in GPP largely occurring in the rainy season due to the solar radiation and high humidity in these areas. They also compared these results with those of Yang et al.^29^ in the Amazon, where droughts that occurred in this period created a less productive albeit greener forest. If significant droughts continue to occur within a five year frequency as previously highlighted and discussed in numerous periodicals, and ENSO events are more frequent and extreme, these biomes may become highly modified in terms of their capacity to sequester atmospheric CO_2_. The biomes would be contributing significant volumes of atmospheric CO_2_ emissions.

Despite the low fire densities in the Caatinga biome, as well as Pantanal and Pampa, there is a need for public policies to be implemented punctually and spatially to facilitate fire prevention and effective fire fighting^30^. Caatinga showed the potential for high alteration, largely in in well preserved areas. In a study on Pernambuco-Brazil that analyzed Land Remote-Sensing Satellite imagery, Dos Santos et al.^31^ found that fragments of dense vegetation were more exposed to degradation and consequent landscape alteration, potentially increasing the burning and resultant carbon emissions in Caatinga. It is worth noting that Caatinga is the most populated biome and occurs in a drier region of Brazil. As such, in addition to landscape changes as a result of increased fires, the acceleration of fires from climate change may lead to dramatic social consequences^31^.

### The commitment to the Paris Agreement

In the Paris Agreement, Brazil committed to reducing its emissions from 2.1 Gt CO_2_e in 2005 to 1.2 Gt CO_2_e by 2030. To achieve this goal, the country has established a series of targets, including zero illegal deforestation in the Amazon by 2030 (MMA, 2020). When it signed the agreement, Brazil was in a place of confidently being able to achieve these targets, demonstrating a 67.35% reduction in the deforestation rates for the legal Amazon (from 19,014 km^2^ in 2005 to 6207 km^2^ in 2015). However, in 2019 there was a 30% increase in deforestation compared to 2018 (7536 to 9762 km^2^)^2^. This recent increase in deforestation may be attributed to the environmental policies adopted by the new Brazilian government; these policies have promoted a dismantling of the inspection agencies which ultimately enables the occupation and exploitation of the Amazon^12^. Although our results have not shown a recent increase in fire foci and emissions in the evaluated years, these findings are concerning given that the Amazon is responsible for the greatest emissions and there is very little political will to reduce degradation in the future. Other current government measures that have the capacity to exacerbate deforestation and fire foci is the (1) recent release of sugarcane planting in the Amazon and Pantanal, which was previously prohibited^32–34^; and (2) the intention to open up indigenous territories for crop cultivation^35^.

In Cerrado, the biome with the second-largest emissions with portions of its area is within the legal Amazon, a major aspect of concern is soybean cultivation^36^. In the Amazon, there are agreements such as the Soy Moratorium to inhibit deforestation^37^, the Cattle Agreement^38^ and the New Brazilian Forest Code allowing deforestation of only 20% of the property. However, there are no such protective moratoriums for the Cerrado and existing legislation allows deforestation of between 65 and 80% of the property^39^. Soybean is already present in indigenous territories^35^ and its new agricultural frontier is in the Matopiba region, which contains portions of Cerrado in Maranhão, Tocantins, Piauí and Bahia states^40^. This region is where the remaining 20% of preserved Cerrado is concentrated^41^ and which experiences the highest incidences of fire foci (Figs. 5, 6, 7). The Matopiba encompasses 337 municipalities distributed over 73 million hectares that have been converted into soybean and pasture areas^42^. In terms of the relative magnitude of emissions from the Cerrado, in 2016, the emissions were higher than the total emissions from the Brazilian industry sector in the same period^3^.

Another crop that deserves attention is sugarcane; Brazil is the world’s largest producer of sugarcane, with 10,123.5 Mha planted in the 2018/2019 harvest to produce sugar and ethanol^43^. This crop is located mainly within the Atlantic Forest as well as some areas within the Cerrado, in the mid-south region of the country. One of the measures proposed by the Brazilian government to achieve the Paris Agreement commitments was to increase the share of sustainable biofuels in its vehicle fleet to 18% by 2030. For this to happen, the ethanol sector will need to increase its planted area from 1.2 to 5 Mha in the next decade, which will occur at the expense of replacing pastures (72%) and natural vegetation mosaics (19%)^8^. This increase in planting area may lead to greater deforestation and ultimately GHG emissions. There is also concern regarding the harvesting method, which can be manual or automated. In the manual method, sugarcane is burned to fend off venomous animals and facilitate cutting. Each burned hectare emits close to 7591 t CO_2_eq/ha^44^; part of the fire foci in the mid-south region of the country in the Cerrado and Atlantic Forest and the northernmost portion of the Atlantic Forest is due to the harvesting of sugarcane preceded by burning (Fig. 4).

All these factors relating to climate phenomena are counterproductive to Brazil’s success in reaching its emissions reduction commitments. When we analyze the percentage of emissions attributable to "Land and Forest Use Change" in relation to total Brazilian emissions (i.e., from energy, industrial processes, agriculture and waste), we observed that between 2005 and 2018, this component was responsible for 50.15% of emissions^3^. As such, land use changes would be the most promising sector for the Brazilian government to invest in order to reduce its emissions to meet the Paris Agreement commitments.

The autoregressive integrated moving average (ARIMA) modeling for fire foci and carbon emissions showed a significant increase in the former for the Amazon and Cerrado and low carbon emissions in other biomes. These are the two biomes of concern that account for the highest carbon emissions as they are agricultural frontier areas. The Amazon has experienced an increase in deforestation of 43.93% from 2018 to 2019, from 7033.05 to 10,123.17 km^2^, respectively^45^. The Cerrado still has a high conversion rate whilst stabilizing in the last two years, from 6634.09 km^2^ in 2018 to 6483.40 km^2^ in 2019^46^. The modeling projects that the total gross emissions from all Brazilian biomes will be 5.7 Gt CO_2_ in 2030 and, more importantly, it shows no signs of decrease throughout the decade. A decrease is required for Brazil to at least approach the emission reduction commitments under the Paris Agreement.

Deforestation associated with fires and emissions in the Amazon and Cerrado will compromise Brazil’s ability to achieve these targets for 2030. Brazil has already shown that it is possible to reduce deforestation. In the 2000s, the country experienced a 70% reduction in the deforestation rate within the Amazon, attributed to the enforcement of laws, interventions in soy and beef supply chains, restrictions on access to credit, and the expansion of protected areas^10^. The prevention of deforestation in the Amazon will require significant political effort by the current Brazilian government and national congress representing the interests of agribusiness. This same effort is also required for the Cerrado, which urgently needs a moratorium for zero deforestation to preserve its last continuous and untouched wilderness areas that are being converted into pasture, soybean and sugarcane^40,41^.

We detected a significant increase in the fire foci for the Atlantic Forest, Cerrado, Pantanal and Pampa biomes. The Amazon and the Cerrado were biomes that had the highest burned areas and emitted the most carbon between 1999 and 2018. The application of the ARIMA model to the fire foci and carbon emissions time series from 1999 to 2018 satisfactorily expressed the evolution of these factors for all Brazilian biomes. In the forecasted time series, a significant increase in fire foci was observed for all Brazilian biomes for 2019–2030. Our results show that Brazil will continue to play a prominent role on the global stage with regard to its contribution to global warming. The continuity of deforestation in the Amazon and Cerrado associated with high carbon and CO_2_ emissions will compromise the country’s ability to achieve the emissions reduction targets that it had previously committed to under the Paris Agreement. Deforestation in the Brazilian Amazon between January and June in 2020 was 46% higher than the four year average over the same period from 2016 to 2019^47^. Although we have not evaluated the 2019 and 2020, the impact of new environmental policies adopted by the current Brazilian government is evident. These policies have allowed for the abrupt reversal of the previous decrease in fires observed since 2017. Mitigating the impact of these new policies will depend on a new approach by the Brazilian government to address deforestation in agricultural frontier areas. Politicians, entrepreneurs and society as a collective need to engage in a major debate on the directions the country needs to take in order to address the existing issue of Brazil being one of the greatest global GHG emitters.

## Methods

### Study area

The study area encompasses six Brazilian biomes; the Amazon, Atlantic Forest, Cerrado, Caatinga, Pampa, and Pantanal (Fig. 9). These biomes cover the entire country, with the Amazon being the largest biome (49.3%), followed by the Cerrado (23.9%), Atlantic Forest (13.0%), Caatinga (9.9%), Pampa (2.1%), and Pantanal (1.8%)^48^.

### Amazon biome

More than 2500 tree species grow in the Amazon biome, corresponding to one-third of the world’s tropical wood, and more than 30,000 plant species^49^. The Amazon also represents one of the largest drainage basin in the world, covering an area of 6,000,000 km^2^. The most important forests in this biome are the dense ombrophilous forests. The size of the biome corresponds to the area of the states in the North Region (Acre, Amapá, Amazonas, Pará, Rondônia, Roraima, and Tocantins), the state of Mato Grosso and part of the state of Maranhão. According to the Köppen classification, updated by Alvares et al.^50^, the climate type is "A", and may be divided into four main sub-climates; tropical monsoon ("Am"), dry and humid tropical ("Aw"), rainy equatorial ("Af") and hot tropical and wet (“AS”).

### Cerrado biome

The Cerrado has a continuous area covering the states of Goiás, Tocantins, Mato Grosso, Mato Grosso do Sul, Minas Gerais, Bahia, Maranhão, Piauí, Rondônia, Paraná, São Paulo, and the Federal District, in addition to entrances in the Amapá, Roraima and Amazonas states^1^. This biome contains the springs of the three largest hydrographic basins in South America; the Amazon/Tocantins, São Francisco and Prata basins which provide high aquifer potential. According to the Ministerio do meio ambiente^1^, the Brazilian Cerrado is recognized as the richest savanna in the world, with high biodiversity and endemism, supporting 11,627 species of native plants that have previously been cataloged. According to the Köppen climate classification, the prevailing climate of the Cerrado is the tropical seasonal (Aw) with a rainy summer and dry winter. The new classification defined by Alvares et al.^50^ divides the Cerrado into seven climatic sub-types; temperate climate with mild summer (Cfb), subtropical climate with hot summer (Cfa), altitude climate (Cwb), hot semi-arid climate (BSh), humid subtropical climate (Cwa), tropical climate with dry winter (Aw) and humid and sub-humid tropical climate (Am).

### Atlantic forest biome

The Atlantic Forest biome occupies more than 17 Brazilian states, partially extending throughout the Brazilian coast^1^. The forest formations are of the dense ombrophilous, mixed and open type, seasonal semideciduous, and deciduous forests^1^. The Atlantic Forest has about 20,000 plant species (approximately 35% of the total existing species in Brazil), including several endemic and endangered species. According to the Köppen climate classification, the climate is humid tropical, being directly influenced by air masses from the Atlantic Ocean. In the new climate classification proposed by Alvares et al.^50^, this region experiences eight climatic sub-types: temperate climate with mild summer (Cfb), subtropical climate with hot summer (Cfa), altitude climate (Cwb), hot semi-arid climate (BSh), climate subtropical humid (Cwa), tropical climate with dry winter (Aw) and humid tropical climate, sub-humid climate (Am) and humid or super-humid tropical climate (Af).

### Caatinga biome

The Caatinga biome includes the Alagoas, Bahia, Ceará, Maranhão, Pernambuco, Paraíba, Rio Grande do Norte, Piauí, Sergipe, and northern Minas Gerais states^1^. About 27 million people live in the region, most dependent on the natural resources within this biome to survive. Caatinga’s biodiversity supports several economic activities in agroforestry and industry, especially in the pharmaceutical, cosmetics, chemical, and food sectors. Caatinga is dominated by steppe savannah vegetation; this includes vegetation with predominantly low trees and shrubs that generally lose their leaves in the dry period (deciduous species) and many cactus species. The Köppen classification for this region defines the climate of the Caatinga as tropical semiarid. In the Caatinga, there are three climatic sub-types; warm semi-arid climate (BSh), hot desert climate (BWh) and tropical climate with a dry winter (Aw^50^).

### Pampa biome

The Pampa biome has low field vegetation in flat land relief. Its occurrence is restricted to the southern state of Rio Grande do Sul. It has a distinct flora and fauna that has not yet fully been scientifically classified^1^. It holds the most significant portion of the Guarani aquifer. The Köppen climate classification for this biome is a temperate climate, with average temperatures of 18 °C. The new climatic classification defined for this biome is the subtropical climate with hot summers (Cfa)^50^.

### Pantanal biome

The Pantanal biome is the largest wetland in the world. It covers part of the Mato Grosso and Mato Grosso do Sul states, in addition to parts of Bolivia and Paraguay^1^. It contains an important wealth of biological, terrestrial, and aquatic diversity. The vegetation of the Pantanal biome is very diverse, mainly due to flooding and varying soil classes. According to the Köppen climate classification and the update proposed by Alvares et al.^50^, the climate is tropical with dry winter (Aw).

### Historical time series of monthly fire foci and carbon emissions

The monthly data on fire foci and carbon emissions was acquired between 1999 and 2018. Fire foci data were calculated using the MODIS sensor product MCD14DL (TERRA/AQUA). Near real-time (NRT) MODIS thermal anomalies/fire locations—collection 6 was processed by the National Aeronautics and Space Administration’s Land, Atmosphere Near real-time Capability for EOS (LANCE) Fire Information for Resource Management System (FIRMS), using swath products (MOD14/MYD14) rather than the tiled MOD14A1 and MYD14A1 products. The thermal anomalies/active fire represents the center of a 1 km pixel flagged by the MODIS MOD14/MYD14 fire and thermal anomalies algorithm^51^ to contain one or more fires within the pixel. This characteristic is the most basic fire product in which active fires and other thermal anomalies, such as volcanoes, are identified. Data were downloaded directly from FIRMS (https://firms.modaps.eosdis.nasa.gov/) and arranged in the shapefile format (https://earthdata.nasa.gov/active-fire-data). The FIRMS fire map enables interactive browsing of the full archive of global active fire detections from MODIS and Visible Infrared Imaging Radiometer Suite (VIIRS). Near real-time fire data was available within approximately 3 h of the satellite overpass and imagery was available within 4–5 h. The same data generated from Terra/Aqua for fire foci was used from the Brazilian fires database^6^.

Carbon emissions (in Tg) were extracted from the fourth version of the Global Fire Emissions Database^52^. This product provides carbon emissions at a 0.25° spatial resolution and categorizes emissions by the types of fire to calculate gas traces using emission factors (https://www.globalfiredata.org/). All of these datasets are based on burned areas driven by a small burned area. Emissions were computed at a monthly temporal resolution and released at this time, as well as a day cycle based on Mu et al.^53^. Emission groups contained a 12-month dataset (01, 02,…, 12), with calculated carbon in units of g cm^*−*2^ month^*−*1^. Biosphere flows included monthly net primary production (NPP), heterotrophic respiration (Rh), and fire emissions (BB), all expressed in g Tg m^−2^ month^*−*1^.

### ARIMA modeling (past and future) of fire, carbon and CO_2_ emissions

The ARIMA model was used to predict expected probable carbon emissions (in Tg) and the number of fire foci for 2019–2030 was based on a number of changes from the 1999–2018 data time series, representing carbon emissions (Tg) and fire foci (number). ARIMA modeling has been considered a robust model used in numerous scientific fields, in an attempt to predict the future of numerous input variables^54,55^. ARIMA models have two general forms (p, d and q) and (P, D, Q) m, non-seasonal and seasonal, respectively, as shown in Eq. (1). The seasonal model I was used in this study where, AR (p) refers to the number of delay observations included in the model, also referred as the delay order in the regression equation for the Y series. The I (d) refers to the number of times gross observations were differentiated, referred to as the degree of differentiation, and MA (q) is the moving average terms, which leads to the observation of previous errors:

1 $$Y_{{t = c + \emptyset_{1} y_{d} t - 1 + \emptyset_{p} y_{d} t - p + \cdots + \emptyset_{1} e_{t - 1} + \emptyset_{q} e_{t - q} + e_{t} }}$$

where y (d) is Y differentiated d times; c is a constant; p is the autoregressive order; d is the differentiation order (1 or 2 typically); and q is the moving average order.

Validation took place with the generation of the 10-year dataset; that is, from the model the previous series of 140 samples were generated to predict the variables for the following year. All years considered were validated from statistical indicators, such as the Standard Error of Estimation (EPE), coefficient of determination (*R*^2^), and Willmott agreement index (D).

The simulation of projected monthly carbon emissions and fire foci variables was conducted for the 12-year period from January 2019 to December 2030 (total 144 samples), using monthly data from 1999 to 2018. According to Sanderson and Fisher^56^, this type of simulation is essential, as it is able to inform future risks and the events of climate change, such as fire and emissions.

To compare our results with the Paris agreement regarding the observations conducted on CO_2_ emissions in 2005 (2.1 Gt CO_2_) and the target set in the Paris agreement by 2030 (1.2 Gt CO_2_), we transformed carbon emissions into CO_2_ emissions. However, it is already recognized that most carbon emissions occur largely in the form of *CO*_2_. Carbon emission data was converted into CO_2_ mass, assuming that any carbon emitted into the atmosphere would oxidize into CO_2_. Data was converted from "g of C" to "g of CO_2_". For this purpose, we used the ratio between the molecular atomic mass of a CO_2_ molecule (44) and the mass of a carbon atom (12) (i.e., 44/12), obtaining a ratio of approximately 3.7^57–59^. All processing was conducted with the R software version 3.5.1, using the available libraries (MASS, tseries, forecast, readxl, raster, rgdal, maptools, RSAGA and ggplot2)^60^.

### Statistical analysis

Boxplot graphics were constructed for the fire foci and carbon emissions data associated with biomes on a monthly scale. Statistical analysis was based on the Mann–Kendall test (MK)^61,62^ in order to identify significant trends in annual fire foci and carbon emissions. This procedure considers the stability hypothesis of the occurrence of successive and independent values with maintenance of the same probability distribution. Variables were subjected to the Pettitt^63^ non-parametric test, which enables the identification of years with an abrupt change within the time series.

Cluster analysis (CA) was applied to identify biomes with homogeneous distribution of fire foci and carbon emissions overtime^18,64,65^. The Euclidean distance was considered a measurement of dissimilarity^66^, over the calculus of distances between the municipalities. In the grouping of biomes, the complete linkage hierarchical method was used (i.e., complete linkage) as described by Johnson and Wichern^67^ and Mardia et al.^68^. The agglomerative method of complete connection consists of the measure of similarity between two clusters, defined by the most significant distance from any point in the 1st cluster to any point in the 2nd cluster.

All analyses in this study were carried out using R version 3.4.3 software^60^, utilizing the following packages; ggplot2, trend, Kendall and factoExtra.

## Supplementary information

Supplementary Information
