# Supplementary material for: Persistent fire foci in all biomes undermine the Paris Agreement in Brazil
Source: Sci Rep. 2020 Oct 1;10:16246. doi: 10.1038/s41598-020-72571-w (PMC7529887; doi:10.1038/s41598-020-72571-w)
Supplement: Supplementary file 1 — Supplementary Information [file 41598_2020_72571_MOESM1_ESM.docx]

**Supplementary Information**

**Persistent fire foci in all biomes undermine the Paris**

**Agreement in Brazil**

# Carlos Antonio da Silva Junior^1^, Paulo Eduardo Teodoro^2,*^, Rafael Coll Delgado^3^, Larissa Pereira Ribeiro Teodoro^2^, Mendelson Lima^4^, Ariane de Andrea Pantaleão ^2^, Fabio Henrique Rojo Baio^2^, Gileno Brito de Azevedo^2^, Glauce Taís de Oliveira Sousa Azevedo^2^, Guilherme Fernando Capristo-Silva^5^, Damien Arvor^6^, and Cassiele Uliana Facco^7^

^1^State University of Mato Grosso (UNEMAT), Department of Geography, 78555000, Sinop, Mato Grosso, Brazil

^2^Federal University of Mato Grosso do Sul (UFMS), 79560-000, Chapadão do Sul, Mato Grosso do Sul, Brazil

^3^Department of Environmental Sciences, Forest Institute, Federal Rural University of Rio de Janeiro (UFRRJ), 23897-000, Seropédica, Rio de Janeiro, Brazil

^4^State University of Mato Grosso (UNEMAT), 78580000, Alta Floresta, Mato Grosso, Brazil

^5^Postgraduate Program in Agronomy, Federal University of Mato Grosso (UFMT), 78555000, Sinop, Mato Grosso,

Brazil

^6^CNRS, UMR 6554 LETG, Université Rennes 2, 35043 Rennes, France

^7^State University of Mato Grosso (UNEMAT), Postgraduate Program in Management and Regulation of Water Resources - ProfÁgua, Cuiabá, Mato Grosso, Brazil

**
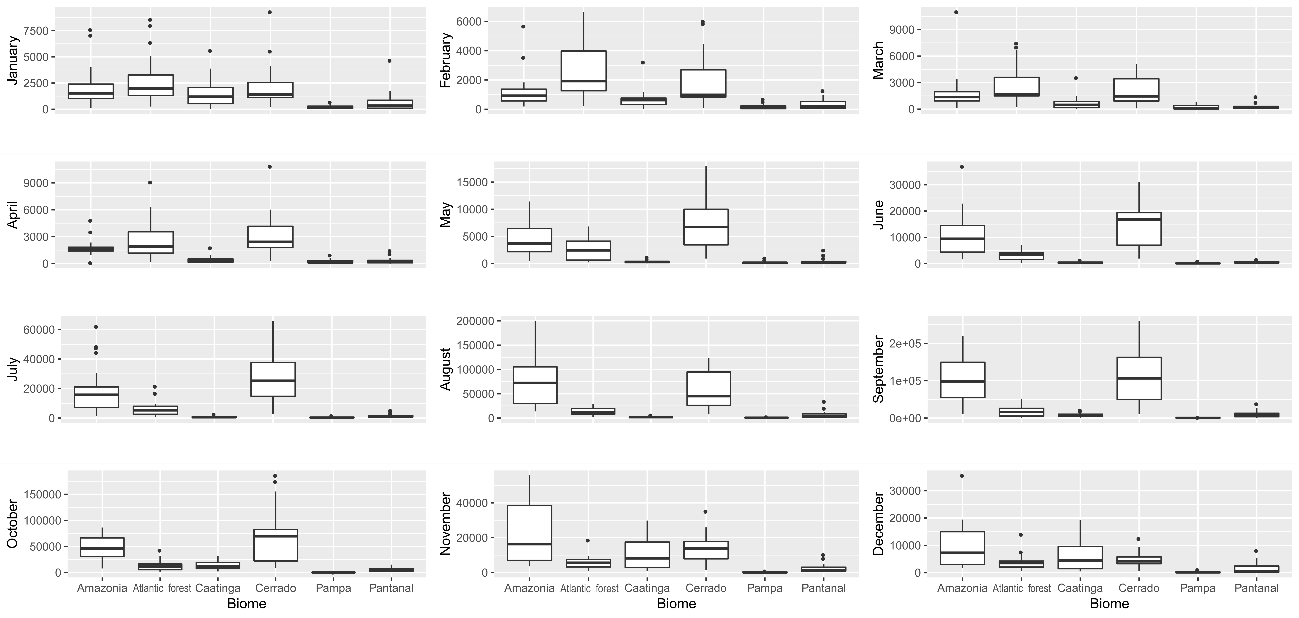
**

**Figure S1.** Boxplot applied to the monthly fire foci (count) between 1999 and 2018.

**
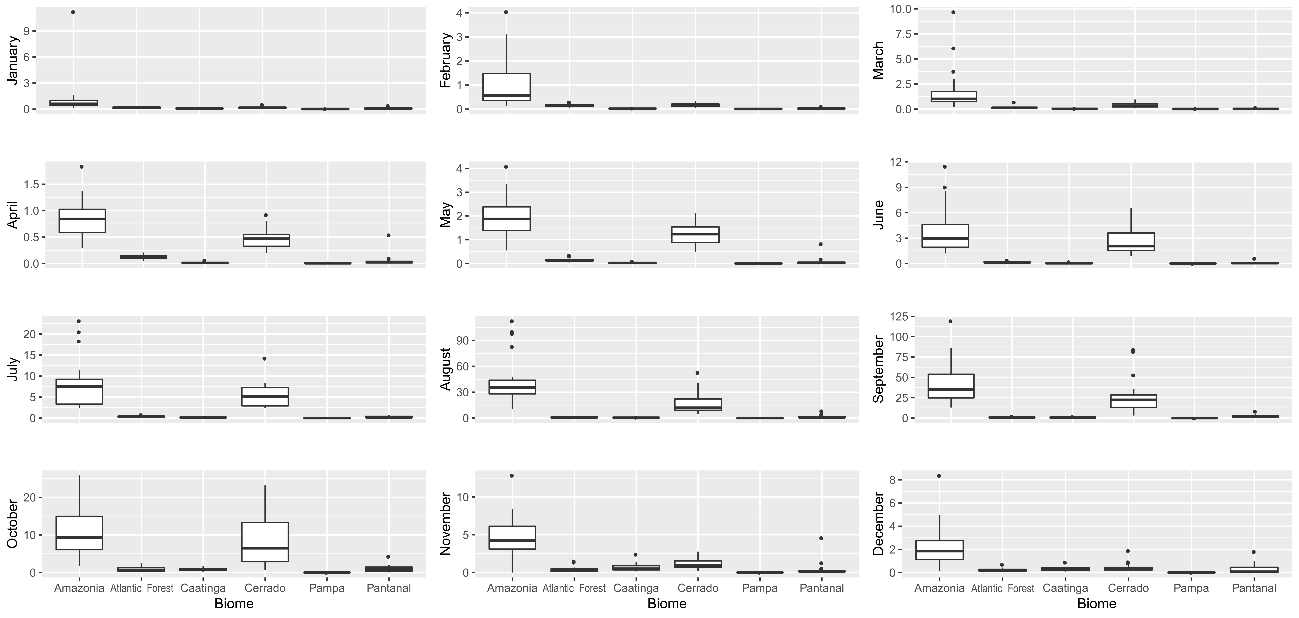
**

**Figure S2.** Boxplot applied to the monthly Carbon emission (Tg) between 1999 and 2018.
